# Self-dilation for therapy-resistant benign esophageal strictures: towards a systematic approach

**DOI:** 10.1007/s00464-018-6037-z

**Published:** 2018-01-18

**Authors:** Emo E. van Halsema, Chantal A. ’t Hoen, Patricia S. de Koning, Wilda D. Rosmolen, Jeanin E. van Hooft, Jacques J. Bergman

**Affiliations:** Department of Gastroenterology & Hepatology, Academic Medical Center, Meibergdreef 9, 1105 AZ Amsterdam, The Netherlands

**Keywords:** Benign esophageal strictures, Esophageal dysphagia, Self-dilation, Endoscopic dilation, Esophagus, Endoscopic therapy

## Abstract

**Background:**

Patients with therapy-resistant benign esophageal strictures (TRBES) suffer from chronic dysphagia and generally require repeated endoscopic dilations. For selected patients, esophageal self-dilation may improve patient’s autonomy and reduce the number of endoscopic dilations. We evaluated the clinical course and outcomes of patients who started esophageal self-dilation at our institution.

**Methods:**

This study was a retrospective case series of patients with TRBES who started esophageal self-dilation between 2012 and 2016 at the Academic Medical Center Amsterdam. To learn self-dilation using Savary-Gilliard bougie dilators, patients visited the outpatient clinic on a weekly basis where they were trained by a dedicated nurse. Endoscopic dilation was continued until patients were able to perform self-bougienage adequately. The primary outcome was the number of endoscopic dilation procedures before and after initiation of self-dilation. Secondary outcomes were technical success, final bougie size, dysphagia scores, and adverse events.

**Results:**

Seventeen patients started with esophageal self-dilation mainly because of therapy-resistant post-surgical (41%) and caustic (35%) strictures. The technical success rate of learning self-bougienage was 94% (16/17). The median number of endoscopic dilation procedures dropped from 17 [interquartile range (IQR) 11–27] procedures during a median period of 9 (IQR 6–36) months to 1.5 (IQR 0–3) procedures after the start of self-dilation (*p* < 0.001). The median follow-up after initiation of self-dilation was 17.6 (IQR 11.5–33.3) months. The final bougie size achieved with self-bougienage had a median diameter of 14 (IQR 13–15) mm. All patients could tolerate solid foods (Ogilvie dysphagia score ≤ 1), making the clinical success rate 94% (16/17). One patient (6%) developed a single episode of hematemesis related to self-bougienage.

**Conclusions:**

In this small case series, esophageal self-dilation was found to be successful 94% of patients when conducted under strict guidance. All patients performing self-bougienage achieved a stable situation where they could tolerate solid foods without the need for endoscopic dilation.

Benign esophageal strictures can have various causes such as post-surgical ischemic strictures, radiotherapy-induced, post-endoscopic dissection, ingestion of caustic substances, reflux-induced, and other rarer causes [1–6]. They can be divided in the simple (short, not angulated, allow passage of endoscope) and complex (angulated, > 2 cm, severely narrowed luminal diameter) strictures [7]. Over 80% of patients with benign esophageal strictures are successfully treated with repeated endoscopic bougie or balloon dilation [5, 8, 9]. However, a subgroup of patients suffer from therapy-resistant benign esophageal strictures (TRBES). Kochman et al. proposed a definition to distinguish two types of resistant strictures: (1) the refractory stricture, that cannot be remediated to a diameter of 14 mm over five endoscopic sessions at 2-week intervals, and (2) the recurrent stricture, in which case a satisfactory luminal diameter cannot be maintained for 4 weeks once the target diameter of 14 mm has been reached [10]. Complex and nonsurgical strictures are more prone to meet this definition [5, 11, 12]. When patients fail to respond to standard repeated dilations, other endoscopic options include the addition of steroid injections and, in case of a suitable morphology, incision of the stricture [13]. Another option is temporary placement of a self-expandable stent, which is effective in approximately 40% of cases [14]. Besides the high risk of recurrent dysphagia, stent migration (29%) and adverse events (21%) are common problems with the use of self-expandable stents for the treatment of benign esophageal strictures [14].

Although reports of patients performing esophageal self-dilation have already been published in the early 60s [15–17], this treatment option is rarely reported in modern literature and only consists of some small case series [18–21]. For selected patients, self-dilation with dilation bougies may allow them to regain autonomy and reduce the need for endoscopic dilations. Case series have reported excellent outcomes with 90% clinical success rates, including 90–100% tolerability and intake of solids in 90–100% of patients without any dilation-related adverse events [18–20]. These results suggest that self-dilation is a valid alternative to repeated endoscopic dilations for a subgroup of patients with TRBES. At our institution, we offer self-dilation to patients with TRBES since 2012. In this study, we aim to evaluate the clinical course and outcomes of our self-dilation patients.

## Materials and methods

In this case series, we retrospectively analyzed the clinical course and outcomes of all patients who started self-dilation at our institution. This study was reviewed by our Medical Ethics Review Committee and did not apply to the Dutch Act ‘Medical Research Involving Human Subjects’ (date of review: June 22, 2016). Since 2012, we offer self-dilation to patients with TRBES. All patients performing esophageal self-dilation are prospectively registered in a database by two specialized nurses (CtH and PdK) involved in the training of these patients. Suitable candidates for self-dilation included patients motivated to learn self-bougienage who had (1) chronic dysphagia because of a benign esophageal stricture requiring multiple endoscopic dilations and (2) a stricture morphology that allowed safe self-bougienage. The presence of a diverticulum, an excentric lumen, tortuous strictures, and strictures within 2 cm of the upper esophageal sphincter were considered relative contraindications for esophageal self-bougienage. Salvage surgery was considered a bridge too far because patients were unfit for or refused major surgery, or because of extensive fibrosis involving the stomach after a chemical burn. The primary outcome of the study was the number of endoscopic dilation procedures after the start of esophageal self-dilation. Secondary outcomes of interest were technical success, time to technical success, clinical success, final bougie diameter, and adverse events related to self-dilation. Technical success was defined as introduction of the bougie on a daily basis below the level of the stricture, as indicated by a taped marker on the bougie. We defined clinical success as patients being able to manage their dysphagia themselves at home without the need of repeated endoscopic dilations and having an Ogilvie dysphagia score of 0 or 1 (Table 1) [22].

**Table 1:** Dysphagia grading according Ogilvie [22]

0	No dysphagia
1	Normal diet avoiding certain foods
2	Semi-solid diet
3	Fluids only
4	Complete dysphagia for even liquids

### Procedures

**Fig. 1:**
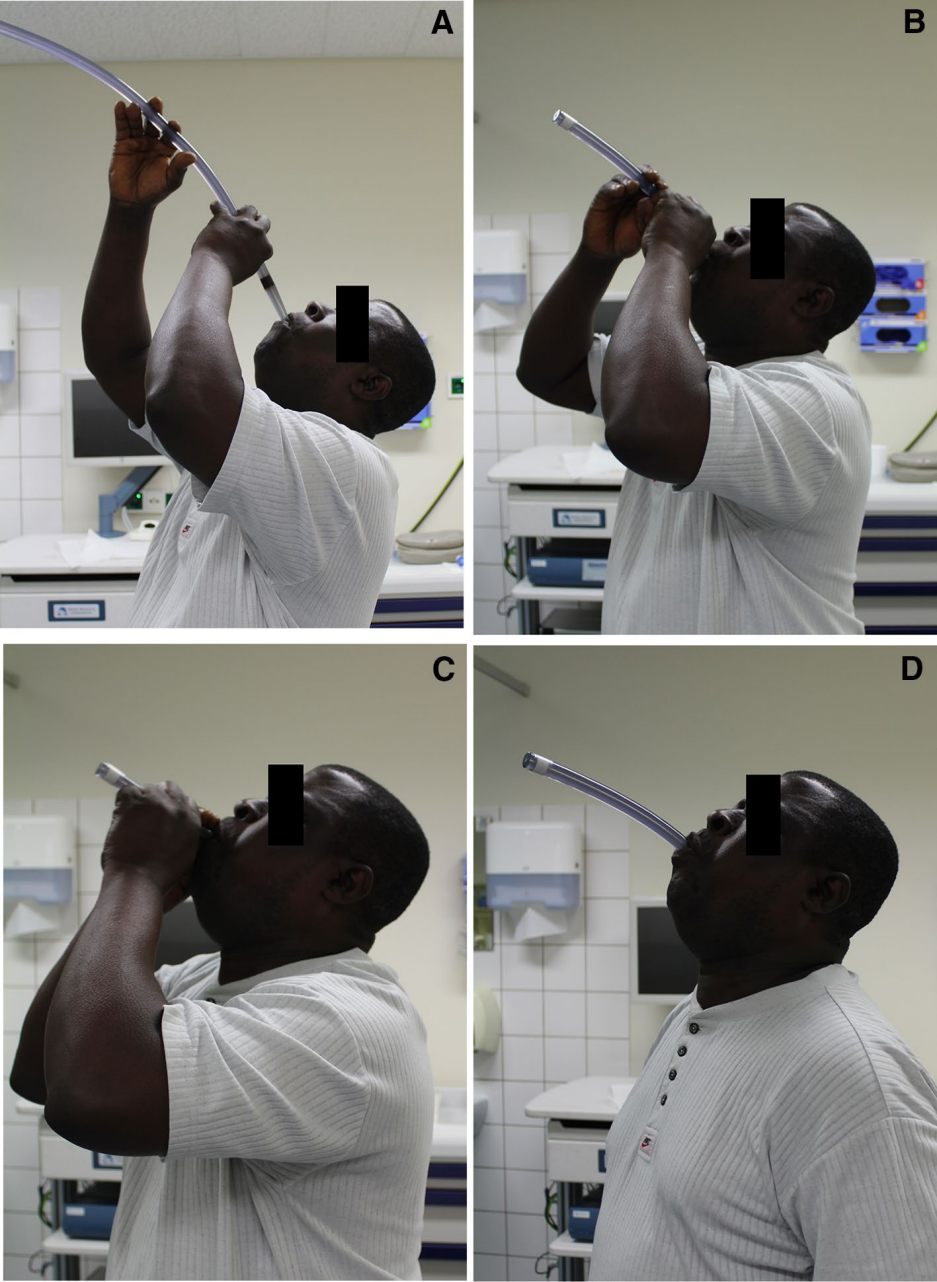
**A–D**. Patient with caustic stricture performing esophageal self-bougienage using a 16 mm Savary bougie dilator

**Fig. 2:**
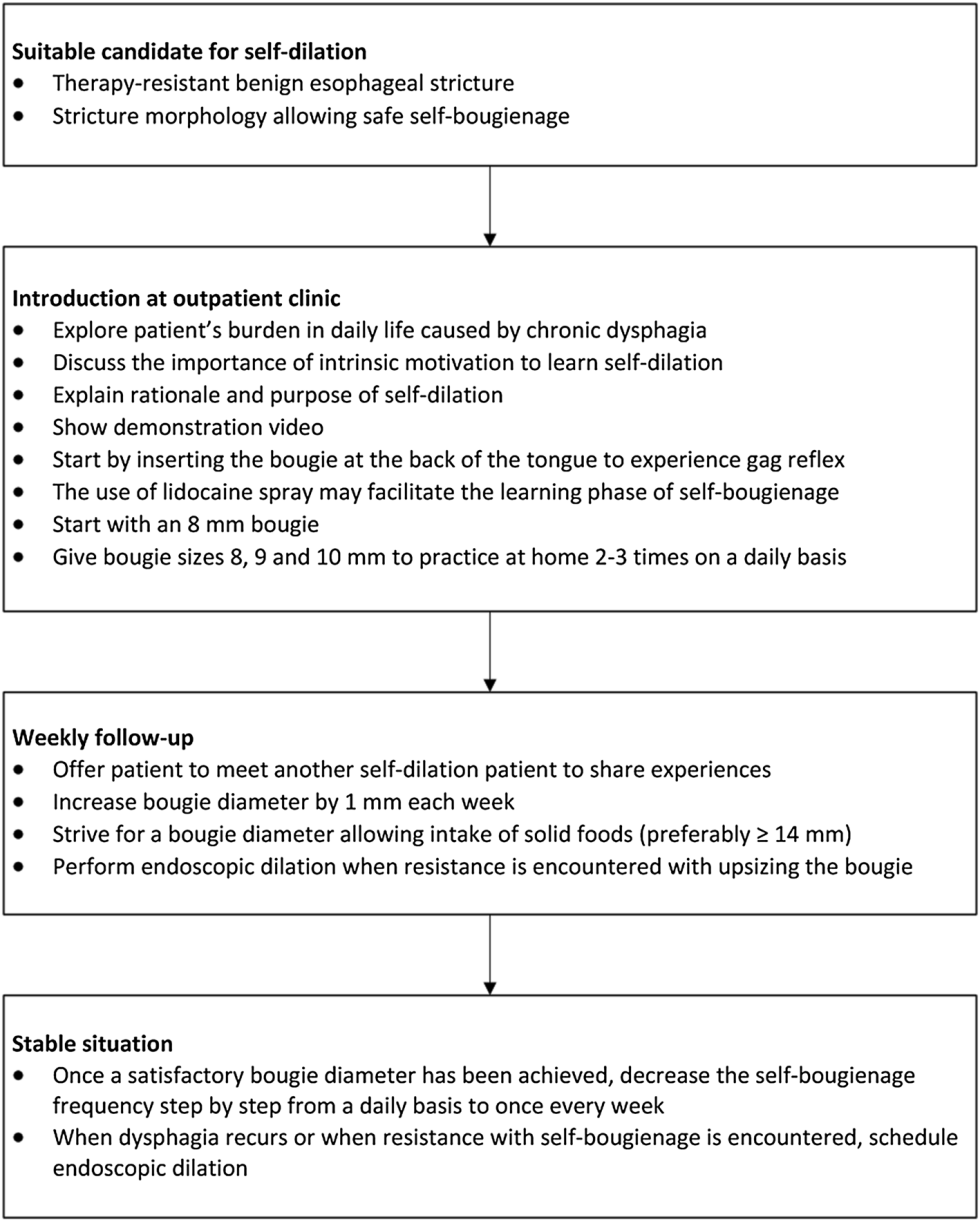
Self-dilation protocol

Patients with therapy-resistant strictures were invited to the outpatient clinic of our specialized nurses, where they were seen on a weekly basis to learn self-dilation using Savary-Gilliard bougie dilators according to the technique as described by Dzeletovic and Fleischer [23]. The first consultation included education about the rationale of self-bougienage, a demonstration video from the Mayo Clinic Arizona, U.S. [24], and contact with another experienced self-dilation patient who could answer to potential questions and concerns. The patient received an 8–10 mm Savary-Gilliard bougie, which was smaller in size than the diameter achieved at the previous endoscopic dilation, to practice self-dilation at home. When the patient was able to demonstrate adequate self-dilation (i.e., technical success) during the next consultation, the bougie was upsized in consecutive steps to a diameter that allowed the patient to tolerate solid foods (Fig. 1). Endoscopic dilations were continued until patients were able to perform self-dilation adequately. Once patients reached a stable bougie diameter by which they could tolerate solid food, the self-dilation frequency was reduced, usually to a frequency of once a week. Whenever dysphagia recurred or patients encountered resistance with self-dilation, an endoscopic dilation was scheduled to relieve complaints and to re-facilitate self-bougienage. See also Fig. 2 for our self-dilation protocol.

### Statistical analysis

Data were retrieved from the electronic medical records. We mainly used descriptive statistics. Analyses were performed on intention-to-treat basis. For the comparison of the number of endoscopic dilations before and after the start of self-dilation, we used the nonparametric Wilcoxon signed-rank test for paired data. Two-sided *p* values < 0.05 were considered statistically significant. We used the statistical software SPSS Statistics version 24 (IBM corp., Armonk, New York, USA).

## Results

Between January 2012 and December 2016, a total of 17 patients started esophageal self-dilation because of TRBES. The stricture etiology was mainly post-surgical (41%) and caustic injury (35%). Strictures were present at multiple levels in the esophagus in 47% of patients with the most dominant stricture mainly located in the proximal esophagus (71%). Before the start of self-dilation, patients underwent a median of 17 endoscopic dilation procedures (IQR 11–27) during a median period of 9 months (IQR 6–36 months). Besides endoscopic bougie or balloon dilations, 47% of patients received additional endoscopic treatments such as steroid injections, incision therapy, or stent placement. The largest bougie size reached with endoscopic dilation had a median diameter of 15 mm (IQR 13–17 mm). Endoscopic treatment was complicated by an iatrogenic perforation in 24% (4/17) of patients, which was managed conservatively in all cases. Further details are summarized in Table 2.

**Table 2:** Baseline characteristics (*N* = 17)

	No. (%)
Gender (male)	10 (59)
Age [median (range)]	65 (32–76) years
Etiology of stricture	
Post-surgical	7 (41)
Caustic	6 (35)
Other^a^	4 (24)
History of esophageal cancer (yes)	8 (47)
Stricture at multiple levels in esophagus (yes)	8 (47)
Stricture longer than 2 cm (yes)	11 (65)
Location of dominant stricture	
Proximal esophagus (< 25 cm from incisors)	12 (71)
Mid esophagus (25–30 cm from incisors)	3 (18)
Distal esophagus (> 30 cm from incisors)	2 (12)
Number of previous endoscopic dilations; median (IQR)	17 (11–27)
Previous endoscopic treatment in addition to bougie/balloon dilation	
None	9 (53)
+ Steroid injections	2 (12)
+ Incision with steroid injections	4 (24)
+ Stent placement	1 (6)
+ Incision and stent placement	1 (6)
Maximum diameter reached with endoscopic dilation^b^; median (IQR)	15 (13–17) mm

*IQR* interquartile range

^a^Peptic (*n* = 1), radiation-induced (*n* = 1) and chronic inflammation of unknown origin (*n* = 2)

^b^Largest bougie size that was passed endoscopically through the stricture

### Self-dilation

The median time from the first endoscopic dilation procedure to the start of self-dilation was 9 months (IQR 6–36 months). The technical success rate of learning self-bougienage was 94% (16/17). A 52-year-old male patient with a 2–3-cm-long post-radiation stricture in the proximal esophagus, who started self-dilation after 24 endoscopic dilation procedures, failed to learn adequate self-dilation because of anxiety and motivational problems. This patient received three additional endoscopic dilations before he was diagnosed with metastasized esophageal carcinoma and died 8 months after the start of self-dilation. The remaining 16 patients were able to perform adequate self-bougienage after a median duration of 16 days (IQR 10–52 days). The median follow-up period from the start of self-dilation was 17.6 months (IQR 11.5–33.3 months).

**Fig. 3:**
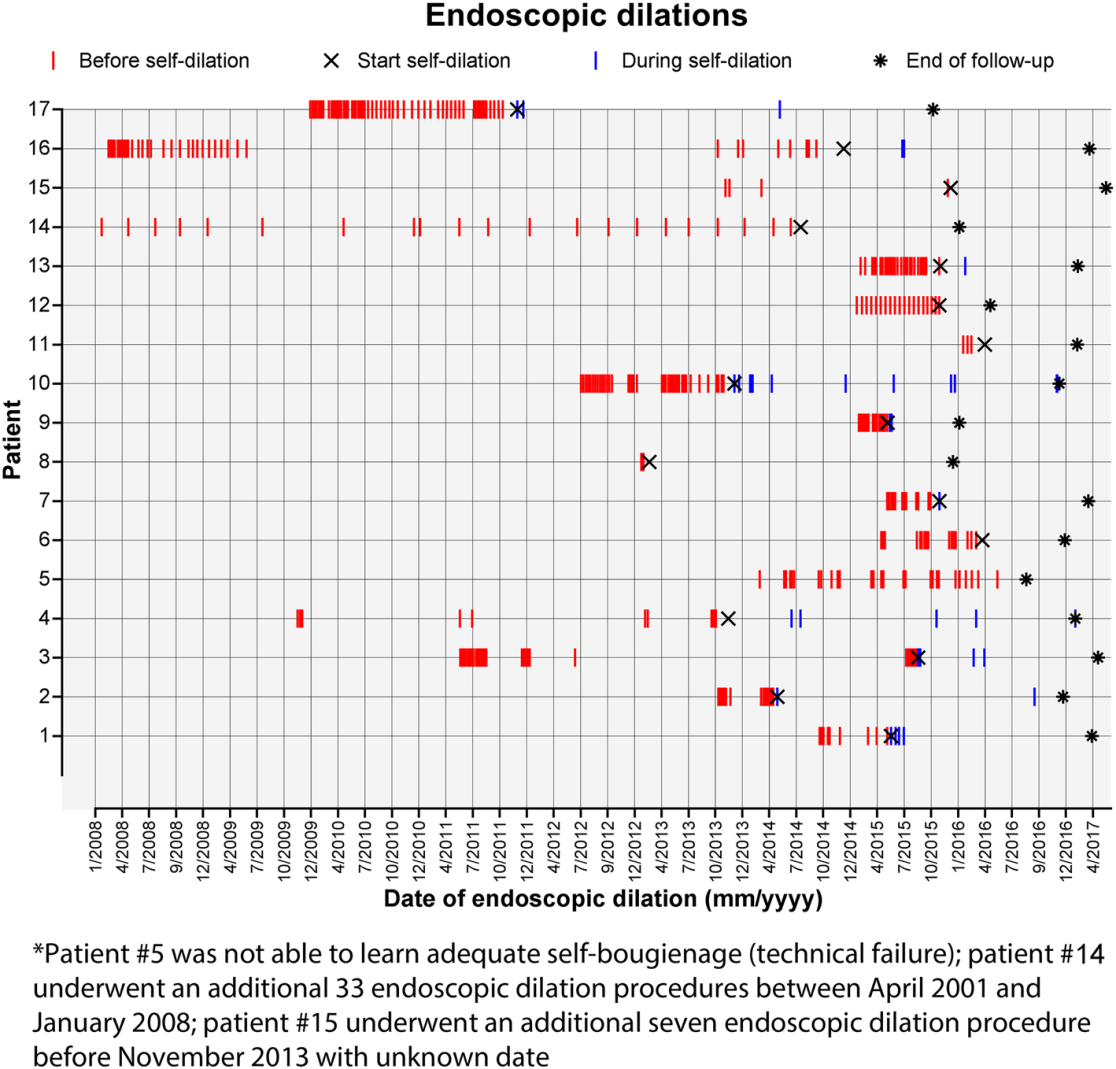
Endoscopic dilation procedures before and after the start of self-dilation*

During the period in which the bougie was upsized to a satisfactory target diameter, 59% (10/17) of patients underwent endoscopic dilation to facilitate the self-bougienage with a median of 1 (IQR 0–2) endoscopic procedure per patient. Once a stable situation was reached with a satisfactory bougie size, 29% (5/17) of patients required additional endoscopic dilation with a median of 0 (IQR 0–1) procedures per patient. The overall number of endoscopic dilation procedures dropped from a median of 17 procedures (IQR 11–27) before the start of self-dilation to a median of 1.5 procedures (IQR 0–3) after the start of self-dilation (*p* < 0.001), see also Fig. 3. The final bougie size achieved with self-bougienage had a median diameter of 14 mm (IQR 13–15 mm). All patients reported that they could tolerate solid foods with a median Ogilvie dysphagia score of 0 (IQR 0–1), making the clinical success rate 94% (16/17). At the end of follow-up, 76% (13/17) of patients were still actively performing self-bougienage, two patients (12%) had stopped self-dilation for a period of 1.5 years and 1.5 months, and two patients (12%) had died because of metastasized esophageal carcinoma. The outcomes are summarized in Table 3.

**Table 3:** Outcomes of esophageal self-dilation (*N* = 17)

	Total
Technical success [no. (%)]	16 (94)
Time to achieve technical success [median (IQR)]	16 (10–52) days
Final bougie size [median (IQR)]	14 (13–15) mm
Duration of follow-up [median (IQR)]	17.6 (11.5–33.3) months
No. of endoscopic dilations after start self-dilation [median (IQR)]	1.5 (0–3)
Able to eat solid foods (Ogilvie dysphagia score ≤ 1) [no. (%)]	16 (94)
Adverse events	
Hematemesis [no. (%)]	1 (6)

*IQR* interquartile range

**Fig. 4:**
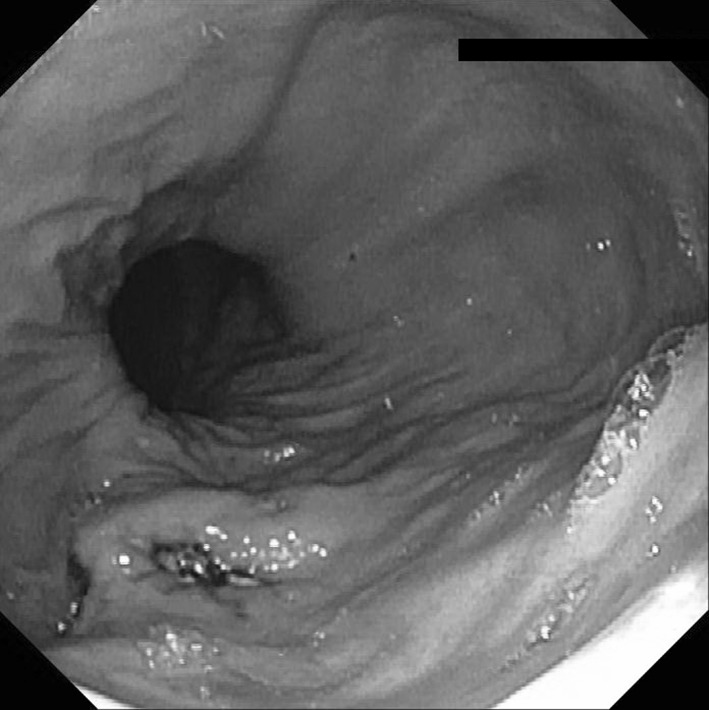
Self-dilation induced small mucosal tear in the gastric cardia in a patient with a hiatal hernia

Regarding the safety of self-bougienage, one patient (6%) presented at the emergency department with hematemesis, no signs of hemodynamic instability and a hemoglobin level of 6.9 mmol/L. Upper endoscopy revealed a small mucosal tear at the gastric cardia (Fig. 4), most likely caused by the tip of the bougie due to too deep insertion with self-bougienage. After careful instructions and marking the bougie with a piece of tape to indicate the appropriate depth of self-bougienage, the patient was discharged on the same day. Another patient was referred to the emergency department because of melena, but upper endoscopy did not show any signs of bleeding. There were no perforations or other serious adverse events related to self-bougienage.

## Discussion

In this small case series of 17 patients with TRBES, 94% of patients were able to learn and perform esophageal self-dilation using bougie dilators. Esophageal self-bougienage led to a statistically significant and, most of all, clinically relevant reduction in the need for endoscopic dilation procedures. All patients performing esophageal self-bougienage reported excellent outcomes regarding dysphagia with all being able to eat and swallow solid foods (Ogilvie dysphagia score ≤ 1). Although the literature on this topic is scarce, other series also support self-dilation as alternative treatment option in patients with TRBES [18, 19, 25]. A case series of 32 patients from the Mayo Clinics, U.S., reported comparable results with a technical success rate of 94% (30/32) and a reduction in the average number of endoscopic dilation procedures from 22 to 1 before and after initiation of self-dilation, respectively, with a median follow-up of 32 months [18]. There was a significant improvement in dysphagia symptoms, as well as in stricture diameter and weight after initiation of self-dilation. No adverse events related to self-bougienage occurred [18].

Besides endoscopic outcomes and dysphagia symptoms, esophageal self-dilation also positively impacts on patient-reported quality of life scores. A study from the University of Michigan, U.S., reported that during a 33-year period 158 patients with cervical esophagogastric anastomotic strictures were taught self-dilation, which was 8% of all patients who underwent a transhiatal esophagectomy during that period [20]. Out of the 78 survivors, 34 patients responded to an esophageal-specific survey, showing that 85% of patients were satisfied or very satisfied with their overall ability to eat and all patients indicated that they would use self-bougienage again under similar circumstances [20]. Patients did not report any adverse events related to self-bougienage using a Maloney dilator with the median duration of self-dilation being almost 10 years [20]. Another series from the Mayo Clinic Arizona found that, when retrospectively assessed by a self-designed questionnaire, global scores for dysphagia and overall quality of life significantly improved under self-dilation compared to the period of endoscopically performed dilations [26]. These results emphasize the positive effect of self-dilation on the patient’s quality of life, including emotional and social well-being compared to hospital bound endoscopic dilations.

Teaching patients how to perform self-bougienage requires strict guidance to overcome anxiety and motivational problems. In our series, one patient who started self-bougienage was not able to insert the bougie below the level of the stricture and stopped further attempts because of anxiety and lack of motivation to continue the training with our nurses. Dzeletovic et al. also reported two failure cases (6%) because of anxiety and in addition three patients (9%) who stopped self-dilation because of intolerance due to throat and/or chest pain [18]. To overcome anxiety, we invited patients on a weekly basis at the outpatient clinic of our dedicated nurses to monitor the progress and answer to questions and concerns. For further motivational support, we occasionally invited other self-dilation patients to the training session so that patients could share their experience. Lidocaine spray or gargle in the bottom of the throat may also be helpful to get through the first phase of self-bougienage [23].

Self-dilation as treatment option in patients with benign esophageal strictures is often unknown or being ignored because of insufficient experience with teaching this technique. However, as demonstrated by our results, self-dilation can be a real solution for a subgroup of patients that do not respond to endoscopic dilation. In our series, 88% (15/17) of patients had a history of at least ten endoscopic dilation procedures. With a target bougie size of 14 mm all patients will be able to eat solid foods and, if desired, patients can even upsize their bougie over 14 mm to achieve a satisfactory situation. The study from the University of Michigan, U.S., showed that after a median duration of almost 10 years 47% (16/34) of patients with a cervical esophagogastric anastomotic stricture had finally stopped self-dilation and the remaining 53% were still performing self-bougienage with an average frequency of once every 2 months [20]. In the series by Dzeletovic et al., consisting of a more heterogeneous population, 10% (3/30) of patients were able to stop self-dilation and 27% (8/30) had decreased the self-bougienage frequency to a maximum of twice weekly [18]. So when a satisfactory bougie size is reached, the self-bougienage frequency can gradually be reduced and thereby patient burden can further be alleviated.

Adverse events related to self-bougienage are rare and particularly perforations have not been reported in the aforementioned case series [18–21]. However, there is a report of a pneumomediastinum related to Eder–Puestow self-dilation [27]. Other rare complications reported are complete swallowing of a Maloney dilator requiring surgical removal from the stomach [28], and repeated unintentional insertion of a Maloney dilator into the right bronchus in a patient with a hypopharyngeal stenosis [29]. So patients should be carefully selected based on a suitable anatomy and stricture morphology, they should be well-informed about the potential risks and considerable resistance with self-bougienage should always be avoided.

This small case series is limited by its retrospective nature, the small sample size, lack of controls, and the selected and heterogeneous population from a single tertiary care center. Nevertheless, this retrospective evaluation shows that self-dilation may be a valid alternative for selected patients with TRBES who require repeated endoscopic dilations. This analysis is the first step to develop a systematic approach for future patients who will start self-dilation at our institution. Future prospective evaluation of clinical and patient-reported outcomes will learn more about the efficacy of self-dilation on the physical, psychological, and social well-being of patients with strictures that hardly respond to endoscopy therapy.

## Abbreviations

TRBES: Therapy-resistant benign esophageal strictures
IQR: Interquartile range
